# Correction to “Unveiling Protist Composition and Diversity Patterns With eDNA Metabarcoding: Comparing Short‐ and Long‐Read Approaches”

**DOI:** 10.1002/ece3.73615

**Published:** 2026-04-29

Skouroliakou, D.‐I., D. W. E. Dupont, Y. Vandenboer, S. D'Hont, K. Sabbe, and I. Schön. 2026. “Unveiling Protist Composition and Diversity Patterns With eDNA Metabarcoding: Comparing Short‐ and Long‐Read Approaches.” *Ecology and Evolution* 16, no. 3: e73218. 10.1002/ece3.73218.

The authors Sofie D'Hont and Koen Sabbe were incorrectly assigned to affiliation (1): IFREMER, DYNECO, Plouzané, France. Their correct affiliation is affiliation (2): Laboratory of Protistology and Aquatic Ecology, Department of Biology, Ghent University, Ghent, Belgium.

The online version of this article has been corrected accordingly.

We apologize for this error.

